# Effect of Bolus Insulin Administration Followed by a Continuous Insulin Infusion on Diabetic Ketoacidosis Management

**DOI:** 10.3390/pharmacy6040129

**Published:** 2018-12-07

**Authors:** Hannah D. Brown, Richard H. Tran, John H. Patka

**Affiliations:** 1 Department of Pharmacy, Novant Health Presbyterian Medical Center, 200 Hawthorne Lane, Charlotte, NC 28204, USA; 2 Site and Patient Access, Pharmaceutical Product Development (PPD), 3900 Paramount Parkway, Morrisville, NC 27560, USA; Richie.tran@ppdi.com; 3 Department of Pharmacy and Drug Information, Grady Health System, 80 Jesse Hill Jr Drive SE, Atlanta, GA 30303, USA; jpatka@gmh.edu

**Keywords:** diabetic ketoacidosis, insulin, bolus, infusion, hyperglycemia

## Abstract

Despite the high incidence of diabetic ketoacidosis (DKA) there is no consensus on the most appropriate way to manage insulin therapy. This study was conducted to evaluate the effect of an insulin bolus on the resolution of DKA. A retrospective chart review of patients admitted between 1 September 2014 and 30 June 2016 with a diagnosis of DKA was conducted. Patients were assigned to the bolus or no bolus group based on provider preference. All patients were initiated on a 0.1 unit/kilogram (kg)/hour (h) intravenous (IV) regular insulin infusion, and patients in the bolus group were treated with a 0.1 unit/kg IV regular insulin bolus. Of the 145 admissions evaluated, 58 received a bolus and 87 did not. There was no difference in baseline demographics, except baseline blood glucose was higher in the bolus group (653 vs. 591 milligrams (mg)/deciliter (dL), *p* = 0.04). The time to resolution of DKA from emergency department admission did not differ between the bolus and no bolus group (15 vs. 15.9 h; *p* = 0.24). There was no difference in total insulin received (1.3 vs. 1.1 units/kg, *p* = 0.18), incidence of hypoglycemia (2 vs. 7%, *p* = 0.64), hypokalemia (16 vs. 29%, *p* = 0.65), or length of hospital stay (3.2 vs. 2.7 days, *p* = 0.27). The insulin bolus administration was not associated with reduced time to resolution of DKA.

## 1. Introduction

Diabetic ketoacidosis (DKA) is a severe complication of diabetes characterized by hyperglycemia, ketonemia, and metabolic acidosis [1,2]. It is caused by an absolute or relative reduction in the amount of circulating insulin, resulting in increased levels of counter-regulatory hormones (glucagon, cortisol, catecholamines, and growth hormone) [1,2]. In DKA, hyperglycemia develops as a result of increased gluconeogenesis and glycogenolysis, as well as impaired glucose utilization by peripheral tissues [1,2]. Free fatty acids are released into circulation from adipose tissue, and are oxidized by the liver into ketone bodies, resulting in ketonemia and acidosis [1,2]. Common precipitating causes of DKA include infection, new-onset diabetes, medication non-compliance, and insufficient insulin therapy [1].

While mortality rates have fallen significantly in recent years, hyperglycemic crises remain a common cause of emergency department visits [2]. In 2014, approximately 207,000 patients in the United States visited the emergency department for a hyperglycemic crisis. Of these, approximately 168,000 patients were hospitalized with a diagnosis of DKA [3]. Despite the high incidence of this disease, there is still controversy regarding its management. Bolus insulin administration is thought to expedite the time to resolution of DKA and decrease overall insulin requirements. However, this has not been demonstrated in clinical trials. In a randomized controlled trial by Kitabchi and colleagues, a high dose insulin infusion (0.14 unit/kilogram (kg)/hour (h) infusion without bolus) was compared to a low dose insulin infusion, with or without a bolus dose of insulin (0.07 unit/kg/h infusion ± 0.07 unit/kg bolus). No difference in time to resolution of DKA was found [4]. Similarly, Goyal and colleagues found no difference in the rate of change of glucose or anion gap, when comparing 0.10–0.16 unit/kg regular insulin bolus to no bolus in patients receiving a 0.1 unit/kg/h regular insulin infusion [5]. In contrast to the benefits, there is a concern that bolus insulin administration may result in hypoglycemia and hypokalemia [4,5]. Goyal and colleagues found that hypoglycemia requiring treatment occurred in 6% of patients, though no statistically significant difference was found compared to a placebo [5]. 

Overall, the current evidence is inconclusive regarding the utility and safety of insulin bolus administration for the initial management of DKA. The 2009 American Diabetes Association consensus statement recommends either a 0.1 unit/kg/intravenous (IV) regular insulin bolus followed by a 0.1 unit/kg/h IV regular insulin infusion, or a 0.14 unit/kg/h IV regular insulin infusion [1]. In contrast, The Joint British Diabetes Societies Inpatient Care Group recommends a fixed rate IV insulin infusion of 0.1 units/kg/h, and advises against the use of an initial insulin bolus in the 2013 guidelines [2]. This study was conducted to evaluate the effect of an insulin bolus, as compared to no bolus, on the management of DKA in patients treated with an insulin infusion.

## 2. Materials and Methods

### 2.1. Study Design

This study was a retrospective chart review of all patients admitted between 1 September 2014 and 30 June 2016, with a diagnosis of DKA as determined by The International Classification of Diseases (ICD)-9/ICD-10 codes. This study was approved by the Grady Research Oversight Committee and was submitted to the Emory University Institutional Review Board (IRB). The Emory University IRB determined this study did not require IRB review or written informed consent (eIRB#: 91962, Date of Decision: 18 October 2016, Deciding Authority: Tracy Cermak, MA, CIP Research Protocol Analyst). Due to its retrospective nature it did not meet the definition of “research” with human subjects, nor “clinical investigation” as set forth in Emory policies and procedures and federal rules. 

### 2.2. Study Setting and Population

This study was conducted at Grady Memorial Hospital, a 953 bed academic medical center and safety net hospital in Atlanta, GA. The hospital sees approximately 130,000 emergency department visits annually, with roughly 270 of the admissions being for DKA. 

Patients were included in this study if they had a primary admission diagnosis of DKA and were between 18 and 89 years of age. Patients were excluded if they did not receive a continuous insulin infusion, if the resolution of DKA did not occur prior to discharge, if the type of diabetes was unknown or unspecified, if the patient had end stage renal disease, was pregnant, or incarcerated (as this is a protected population). Patients were also excluded if a dose outside the institution’s DKA protocol was used for any reason. Treatment assignment was decided by provider preference and therefore could deviate from hospital protocol (example: 0.14 unit/kg/h infusion, or a lower starting dose due to renal insufficiency). 

**Figure 1:**
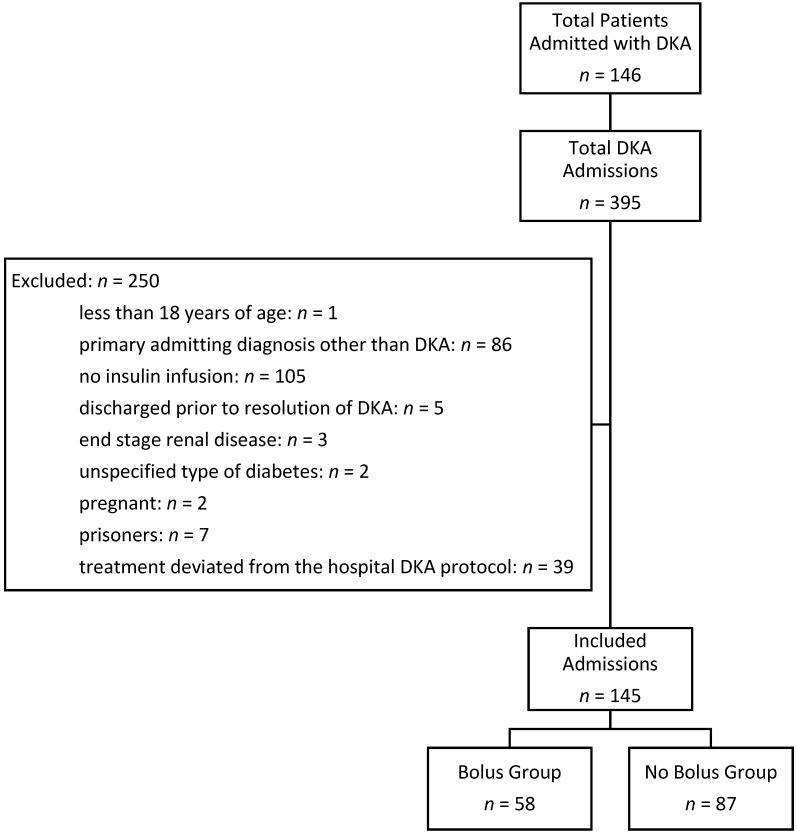
Flow chart of inclusion and exclusion criteria.

A total of 146 patients were admitted 395 times with an ICD-9/ICD-10 diagnosis of DKA between 1 September 2014 and 30 June 2016. Each hospital admission was evaluated separately. A total of 250 admissions were excluded from the analysis: 1 patient was less than 18 years of age, 86 patients had a primary admission diagnosis other than DKA, 105 patients did not receive an insulin infusion, 5 patients were discharged prior to the resolution of DKA, 3 patients were excluded for end stage renal disease, 2 patients were excluded for an unspecified type of diabetes, 2 patients were pregnant, 7 were prisoners, and 39 patients received treatment that deviated from the hospital’s DKA protocol. One hundred and forty five admissions from 76 patients were analyzed. A summary of admissions meeting inclusion and exclusion criteria is presented in Figure 1. 

### 2.3. Study Protocol

All patients were initiated on a 0.1 unit/kg/h IV regular insulin infusion, which was titrated by a nurse-driven protocol. Patients in the insulin bolus group were treated with a 0.1 unit/kg IV regular insulin bolus, prior to the initiation of the insulin infusion. In both groups blood glucose (BG) was monitored every 1–2 h and the infusion rate was adjusted as follows: BG greater than or equal to 400 mg/deciliter (dL), give 8 units of regular insulin IV bolus and increase infusion rate by 1 unit/h; BG 221–399 mg/dL, increase infusion rate by 1 unit/h; and first BG < 220 mg/dL, decrease insulin rate by 50% and change fluids to D5W 0.45% NaCl. Once the BG was below 220 mg/dL it was titrated to maintain a BG between 161 and 220 mg/dL, until DKA resolved. Patients also received IV fluids and electrolyte replacement as necessary.

### 2.4. Outcomes 

The primary endpoint of this study was the time to resolution of DKA from emergency department admission. The resolution of diabetic ketoacidosis was defined as a BG less than 200 mg/dL plus 2 of the following: pH greater than 7.3, anion gap less than or equal to 12 milliequivalent (mEq)/liter (L), and bicarbonate greater than or equal to 15 mEq/L. Secondary endpoints include: time to resolution of DKA from insulin infusion initiation, total insulin received, incidence of hypoglycemia (BG less than 70 mg/dL), incidence of hypokalemia (serum potassium less than 4 mEq/L), survival to hospital discharge, and length of hospitalization. A subgroup analysis was conducted in patients weighing >100 kg to assess the effect of higher insulin doses, in units, on time to resolution of DKA and adverse events. No previous studies have evaluated the impact of body weight on DKA treatment outcomes and a body weight of 100 kg was chosen arbitrarily. 

### 2.5. Data Analysis

Continuous data is reported as the median and range, unless otherwise noted. Patient characteristics between groups were compared using the Fisher’s exact test for categorical variables. Continuous variables were non-parametric and were assessed using the Wilcoxon Rank Sum Test. The Shapiro–Wilk test was used to test for normality. Due to the retrospective nature of the study, data are not likely missing at random. There were 8 missing venous pH values (5 for bolus, 3 for no bolus), 8 missing beta hydroxybutyrate values (3 bolus, 5 no bolus), and 38 missing hemoglobin A1c values (14 bolus, 24 no bolus). A complete case analysis was used to handle missing data. All statistical tests were conducted at the 0.05 significance level. SAS Version 9.4 (SAS Institute Inc., Cary, NC, USA) was used for all analyses.

## 3. Results

Of the 145 admissions evaluated, there were 58 instances of patients receiving an insulin bolus and 87 instances of patients being treated without a bolus. The patients were a median age of 45 years, 46% were female, and 82% were black. Ninety-three percent of patients had a known diagnosis of diabetes, 50% of patients had type 1 diabetes, and the median hemoglobin A1c was 12.7%. Twenty-six percent of patients were admitted to the intensive care unit (ICU). There was no statistically significant difference in baseline demographics, except that the baseline blood glucose was higher in the bolus insulin group (653 vs. 591 mg/dL, *p* = 0.04). The baseline demographics are presented in Table 1.

The primary outcome and time to resolution of DKA from emergency department admission, did not differ significantly between the insulin bolus and no bolus group (15 vs. 15.9 h, respectively; *p* = 0.24). Similarly, there was no difference in the secondary outcome, time to resolution of DKA from insulin infusion initiation between the insulin bolus, and no bolus group (10.8 vs. 11.3 h, respectively; *p* = 0.97). Bolus insulin administration did not result in a significant difference in the total insulin received (*p* = 0.18). Receiving an insulin bolus did not result in a higher incidence of hypoglycemia (*p* = 0.64) or hypokalemia (*p* = 0.65) within 4 h of DKA protocol initiation. No difference in length of hospital stay (*p* = 0.27) or survival to hospital discharge (*p* = 0.40) was observed. Primary and secondary outcomes are presented in Table 2.

These results are consistent with the subgroup analysis of patients weighing >100 kg. In the 17 patients weighing >100 kg, there was no difference in the time to resolution of DKA from admission (*p* = 0.09), time to resolution of DKA from insulin infusion initiation (*p* = 0.09), or the incidence of hypokalemia (*p* = 0.26). No patients weighing >100 kg experienced hypoglycemia. Furthermore, there was no difference in the rates of adverse events between patients weighing <100 kg or >100 kg (hypoglycemia *p* = 1.00, hypokalemia *p* = 0.51). The results are presented in Table 3.

## 4. Discussion

Insulin, in combination with fluid and electrolyte management, is the mainstay of DKA treatment [1,2]. Traditionally, an insulin bolus has been administered to overcome the insulin resistance seen during a hyperglycemic crisis, but in more recent years this practice has come into question, given the concern for hypokalemia and hypoglycemia [6,7,8]. 

In 2008, a small randomized controlled trial compared a high dose insulin infusion (0.14 unit/kg/h infusion without bolus) to a low dose insulin infusion, with or without a bolus dose of insulin (0.07 unit/kg/h infusion ± 0.07 unit/kg bolus) in 37 patients with DKA. Although the dose utilized in this trial differed from the present study, the findings are consistent between the two studies. No difference in time to resolution of DKA, length of hospitalization, or incidence of hypokalemia was found. The study by Kitabchi and colleagues is the only prospective randomized trial assessing the need for bolus insulin administration in adults, but the results were inconclusive given the small sample size, open label design, and imbalanced baseline characteristics between treatment arms [3]. 

In 2010, Goyal and colleagues completed a prospective observational study of 157 patients, comparing a 0.10–0.16 unit/kg regular insulin bolus to no bolus in patients receiving a 0.1 unit/kg/h regular insulin infusion. The treatment groups and baseline demographics were similar to the present study, as were the results. There was no statistically significant difference in the rate of hypoglycemia, rate of change in blood glucose or anion gap, or length of hospital stay [3]. The study was limited by an observational design and the use of 50% dextrose administration as a surrogate endpoint for hypoglycemia [4].

The lack of benefit demonstrated in these trials is consistent with the literature evaluating bolus insulin administration in the pediatric population. In 1980, Fort and colleagues evaluated the effect of bolus insulin administration prior to the initiation of a 0.1 unit/kg/h regular insulin infusion on time to normoglycemia. They found that bolus insulin therapy increased the rate of BG decline during the first hour after administration, but there was no difference in the time to normoglycemia [9]. Similarly, in 1989 Lindsay and Bolte conducted a randomized controlled trial to assess the effect of bolus insulin administration prior to an insulin infusion in 56 episodes of DKA, among 38 pediatric patients. They found that bolus insulin administration did not result in a decreased time to BG less than 250 mg/dL or the total duration of insulin therapy. Both studies were limited by their small sample size [10].

Though retrospective in nature, this study provided additional evidence that bolus insulin administration does not reduce the time to resolution of DKA. While each individual study is insufficient, due to limitations in the study design, to form a conclusive recommendation, together the uniform results suggest that the routine administration of an insulin bolus offers no benefit in the management of DKA. The lack of benefit observed may be the result of the rapid time to reach therapeutic insulin concentrations following the initiation of an insulin infusion. When no loading dose was given, Kitabachi and colleagues found that therapeutic insulin concentrations were achieved within approximately 15 min when a 0.7 unit/kg/h infusion was used, and in under 5 min when a 0.14 unit/kg/h infusion was used [4]. The slightly faster time to therapeutic insulin concentrations appears to be of minimal clinical significance. 

### Limitations

A limitation of this study was the non-randomized cohort design. Group assignments were determined by provider preference, and therefore the presence of confounding variables could not be ruled out. However, the baseline characteristics were well matched between the two groups. The baseline blood glucose was higher in the insulin bolus group (653 vs. 591 mg/dL, *p* = 0.04) which may result in a faster time to resolution of DKA, but the clinical significance of this difference is unclear. Additionally, more patients in the non-bolus group received subcutaneous insulin prior to DKA protocol initiation; however, this did not meet statistical significance. Subcutaneous insulin has shown potential as an alternative route of insulin administration for the treatment of DKA, potentially confounding our results [11,12]. However, when patients who received subcutaneous insulin prior to IV insulin administration were excluded, there was no significant difference in any of the clinical outcomes. The retrospective study design introduces the potential for inaccurate record-keeping and missing data. However, the lab values in this study were routinely recorded as part of DKA management and there was no missing data for any of the primary or secondary endpoints. Another limitation of this study was that multiple admissions were included for some patients. Multiple admissions were analyzed for each patient to increase sample size, as the impact of treatment in one admission is thought to have minimal impact on the outcomes of another admission. However, this practice could result in observations from the same patient being multiplied and influencing the results of the study. An additional limitation of this study was that the determination of the primary outcome, time to resolution of DKA, was based on laboratory values that were not collected at standardized time points. Therefore, it is possible that DKA resolution occurred prior to labs being collected, resulting in a falsely prolonged time to resolution.

## 5. Conclusions

In this non-randomized retrospective chart review, administration of an insulin bolus was not associated with a reduced time to resolution of DKA, when compared to patients not administered an insulin bolus. Bolus insulin administration was not associated with an increased incidence of hypoglycemia or hypokalemia. 

## Figures and Tables

**Table 1:** Baseline demographics.

	Insulin Bolus (*n* = 58)	No Insulin Bolus (*n* = 87)	*p*-Value
**Age (years):** median (range)	43 (21–76)	46 (20–86)	0.94
**Female:** *n* (%)	27 (47)	39 (45)	0.86
**Race:** *n* (%)			0.41
Black	46 (79)	73 (84)	
White	11 (19)	14 (16)	
Hispanic	1 (2)	0 (0)	
**Weight (kg):** median (range)	67 (36–153)	66 (36–128)	0.63
**Diagnosis of Diabetes Prior to Admission:** *n* (%)	52 (90)	83 (95)	0.19
**Type of Diabetes:** *n* (%)			1.00
1	29 (50)	43 (49)	
2	29 (50)	44 (51)	
**Hemoglobin A1c (%):** median (range)	13.2 (7.7–18.8)	12.2 (7.3–17.5)	0.07
**Blood Glucose (mg/dL):** median (range)	653 (305–1717)	591 (234–1546)	0.04
**Anion Gap (mEq/L):** median (range)	27 (9–45)	25 (8–41)	0.37
**Bicarbonate (mEq/L):** median (range)	13 (3–31)	13 (2–32)	0.87
**Potassium (mEq/L):** median (range)	5.2 (3.4–7.8)	5.1 (3.5–8.0)	0.80
**Venous pH (mmol/L):** median (range)	7.20 (7.00–7.50)	7.19 (6.70–7.40)	0.57
**Beta Hydroxybutyrate (mmol/L):** median (range)	8.58 (0.56–9.00)	7.89 (0.18–9.00)	0.40
**Subcutaneous Insulin Prior to Protocol Initiation:** *n* (%)	7 (12)	20 (23)	0.19
**Intensive Care Unit (ICU) Admission:** *n* (%)	18 (31)	20 (23)	0.33

Abbreviations: kg = kilograms, mg = milligram, mEq = milliequivalent, dL = deciliter, L = liter, mmol = millimole. Categorical variables were compared using the Fisher’s exact test and continuous variables were assessed using the Wilcoxon Rank Sum Test.

**Table 2:** Primary and secondary outcomes.

	Insulin Bolus (*n* = 58)	No Insulin Bolus (*n* = 87)	*p*-Value
**Time to Resolution of Diabetic Ketoacidosis (DKA) from Admission (h):** median (range)	15 (1–75)	15.9 (5–154)	0.24
**Time to Resolution of DKA from Insulin Infusion Initiation (h):** median (range)	10.8 (2–72)	11.3 (2–151)	0.97
**Total Insulin Received (units/kg):** median (range)	1.3 (0.3–7.8)	1.1 (0.1–8.3)	0.18
**Hypoglycemia:** *n* (%)	1 (2)	4 (7)	0.64
**Hypokalemia:** *n* (%)	9 (16)	17 (29)	0.65
**Length of Hospital Stay (days):** median (range)	3.2 (0.7–25.2)	2.7 (1.0–20.9)	0.27
**Alive at Hospital Discharge:** *n* (%)	57 (98)	87 (100)	0.40

Abbreviations: h = hours, kg = kilograms. Categorical variables were compared using the Fisher’s exact test and continuous variables were assessed using the Wilcoxon Rank Sum Test.

**Table 3:** Time to resolution of DKA and incidence of adverse events in patients >100 kg.

	Insulin Bolus (*n* = 5)	No Insulin Bolus (*n* = 12)	*p*-Value
**Time to Resolution of DKA from Admission (h):** median (range)	27.0 (9–33)	17.9 (10–154)	0.09
**Time to Resolution of DKA from Insulin Infusion Initiation (h):** median (range)	23.2 (7–27)	12.2 (6–151)	0.09
**Total Insulin Received (units/kg):** median (range)	3.9 (0.3–7.8)	1.2 (0.5–5.8)	0.5
**Hypoglycemia:** *n* (%)	0 (0)	0 (0)	1
**Hypokalemia:** *n* (%)	0 (0)	4 (33)	0.26
**Length of Hospital Stay (days):** median (range)	2.6 (2.2–9.4)	3.0 (1.4–20)	0.72
**Alive at Hospital Discharge:** *n* (%)	5 (100)	12 (100)	1

Abbreviations: h = hours, kg = kilograms. Categorical variables were compared using the Fisher’s exact test and continuous variables were assessed using the Wilcoxon Rank Sum Test.
